# Structural Investigation for Optimization of Anthranilic Acid Derivatives as Partial FXR Agonists by *in Silico* Approaches

**DOI:** 10.3390/ijms17040536

**Published:** 2016-04-08

**Authors:** Meimei Chen, Xuemei Yang, Xinmei Lai, Jie Kang, Huijuan Gan, Yuxing Gao

**Affiliations:** 1 College of Traditional Chinese Medicine, Fujian University of Traditional Chinese Medicine, Fuzhou 350122, China; mei_tcm@163.com (X.Y.); keerer1990@163.com (X.L.); meinuohua2007@163.com (J.K.); ghjzyz@163.com (H.G.); 2 College of Chemistry and Chemical Engineering, Xiamen University, Xiamen 361005, China; gaoxingchem@xmu.edu.cn

**Keywords:** QSAR, docking, FXR, anthranilic acid derivatives

## Abstract

In this paper, a three level *in silico* approach was applied to investigate some important structural and physicochemical aspects of a series of anthranilic acid derivatives (AAD) newly identified as potent partial farnesoid X receptor (FXR) agonists. Initially, both two and three-dimensional quantitative structure activity relationship (2D- and 3D-QSAR) studies were performed based on such AAD by a stepwise technology combined with multiple linear regression and comparative molecular field analysis. The obtained 2D-QSAR model gave a high predictive ability (*R*^2^_train_ = 0.935, *R*^2^_test_ = 0.902, *Q*^2^_LOO_ = 0.899). It also uncovered that number of rotatable single bonds (b_rotN), relative negative partial charges (RPC^−^), oprea's lead-like (opr_leadlike), subdivided van der Waal’s surface area (SlogP_VSA2) and accessible surface area (ASA) were important features in defining activity. Additionally, the derived3D-QSAR model presented a higher predictive ability (*R*^2^_train_ = 0.944, *R*^2^_test_ = 0.892, *Q*^2^_LOO_ = 0.802). Meanwhile, the derived contour maps from the 3D-QSAR model revealed the significant structural features (steric and electronic effects) required for improving FXR agonist activity. Finally, nine newly designed AAD with higher predicted EC_50_ values than the known template compound were docked into the FXR active site. The excellent molecular binding patterns of these molecules also suggested that they can be robust and potent partial FXR agonists in agreement with the QSAR results. Overall, these derived models may help to identify and design novel AAD with better FXR agonist activity.

## 1. Introduction

Farnesoid X receptor (FXR) is a nuclear receptor expressed in liver, gall bladder, intestine, kidney, and adrenal glands. It regulates important physiological roles in various metabolic pathways involved in bile acid, triglyceride, and glucose homeostasis. Now, FXR has become an attractive target for treating a wide range of metabolic diseases, including diabetes, cholestasis, liver fibrosis, and inflammatory bowel diseases [1,2,3]. Therefore, a number of synthetic steroidal and nonsteroidal FXR agonists have been developed so far. 6-ethyl-chenodeoxycholic acid20 (6-ECDCA) and GW4064 were the most important and widely used steroidal and nonsteroidal FXR agonists [4]. They both constitute full FXR agonists with low nanomolar EC_50_ values of 85 nM and 0.9 M in a reporter gene assay [5], respectively. In addition, a recent study indicated that GW4064 was active on several off-targets [6]. Considering that metabolic diseases require a stable long-term therapy, well tolerated and low toxicity FXR agonists are predominantly required that can be applied over long time. Moreover, full activation of a ligand activated transcription factor may cause many side effects in long-term treatment [7]. Therefore, new potent partial FXR agonists aimed at providing a stable long-term therapy for metabolic diseases have attracted more and more attention nowadays.

Recently, a novel series of partial FXR agonists based on anthranilic acid skeleton have been reported by Merk *et al.* [8,9]. The continued interest in the development of more potent partial FXR agonists prompted us to explore the relationship between structures of AAD and FXR agonist activity. Here, quantitative structure activity relationship (QSAR) methods were introduced to guide lead optimization and study the action mechanism for partial FXR agonists. In this QSAR method, the bioactivity of compounds can be predicted by a mathematical model between physicochemical properties and bioactivity. The mathematical model can be achieved by many general algorithms such as linear and nonlinear algorithms or other new methods such as the spectral-structure activity relationship algorithm [10,11]. This QSAR method has become very useful and is widely applied in many fields for predicting compound properties [12,13], including physical property prediction, biological activity prediction, and toxicity prediction.

To the best of our knowledge, no QSAR study has yet been reported in AAD as FXR agonists so far. In this paper, we attempted to investigate the significant structural and physicochemical features required for improving biological activity and to obtain highly predictive 2D- and 3D-QSAR models so as to assist in the design of new potent partial FXR agonists. Firstly, a stepwise technology combined with multiple linear regression (MLR) was applied to develop predictive 2D-QSAR models for uncovering physicochemical features on FXR activity of AAD. Subsequently, a 3D-QSAR study was also performed to obtain more understanding with respect to chemical structures and biological activity using comparative molecular field analysis (CoMFA). The CoMFA model can provide identification of regions in space where the interactive fields may influence the biological activities in the form of contour maps, which would generate graphical visualization of crucial steric and electrostatic features in 3D Cartesian space [14]. Finally, some important observations were also made during the study concerning nine newly designed AAD with high predicted bioactivity and their interactions with the FXR active site by molecular docking. Molecular docking aims to predict the binding-conformation of ligands to the appropriate target binding site. The success of a docking program depends on two components: the search algorithm and the scoring function. A variety of conformational search strategies have been reported such as systematic or stochastic search or genetic algorithms or simplified molecular input line entry system conformation [15]. Most scoring functions are physics-based molecular mechanics force fields that estimate the energy of the pose within the binding site.

## 2. Results and Discussion

### 2.1. Two-Dimensional Quantitative Structure Activity Relationship (2D-QSAR) Models

#### 2.1.1. Multiple Linear Regression Modeling

After stepwise multiple linear regression (SW-MLR) was performed, the best linear model was generated with five molecular descriptors. The obtained MLR model was given as follows:

pEC_50_ = (0.016 ± 0.002)ASA + (14.001 ± 4.041)RPC^−^ − (0.049 ± 0.0175)SlogP_VSA2 + (0.362 ± 0.158)b_rotN + (0.318 ± 0.276)opr_leadlike − (9.717 ± 2.221)

**Figure 1:**
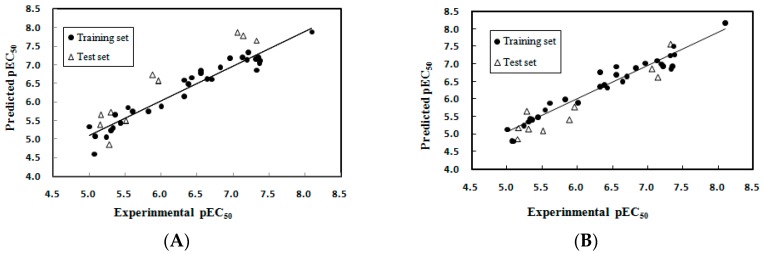
Plots of experimental against predicted pEC_50_ values by (**A**) multiple linear regression (MLR) and (**B**) CoMFA models.

*N*_train_ = 31, *R*^2^_train_ = 0.935, *F*_train_ = 72.353 > *F*_0.005(5,25)_ = 4.43 (the cut off value of *F* distribution), *RMSE*_train_ = 0.219, *Q*^2^_LOO_ = 0.899, RMSE_LOO_ = 0.299, *N*_test_ = 10, *R*^2^_test_ = 0.902, *RMSE*_test_ = 0.534.

where, *N*_train_ and *N*_test_ are the number of compounds in the training set and the test set, respectively. *R*^2^_train_ and *R*^2^_test_ are the squared correlation coefficient of training set and test set, respectively; *Q*^2^_LOO_ is the leave-one-out (LOO) cross-validation squared correlation coefficient; *F* is the *F*-test value; *RMSE* is root mean standard error. The selected variables and their chemical meanings, standard coefficients are shown in Table 1. A variable inflation factor (VIF) (VIF = 1/(1 − *R*_j_^2^), *R*_j_^2^ represents the multiple correlation coefficient of one descriptor’s effect on the remaining molecular descriptors) was calculated to determine if multicollinearity existed among the descriptors in models. If VIF arrays from 1.0 to 5.0, the linked equation is suitable [16]. As shown in Table 1, the VIF of all descriptors were smaller than 4, indicating that the generated model possessed statistic significance and good stability. Table 2 shows the correlation matrix of the selected descriptors. From this table, it can be seen that the linear correlation coefficient value for each pair of descriptors was smaller than 0.85, suggesting that the selected descriptors were independent, meeting the important criterion for the model selections [17]. The predicted results of the MLR model are given in Table 3 and shown in Figure 1A. As described in Table 4, obviously, the MLR model was very successfully built with statistical significance and good prediction ability. The *R*^2^_train_ value of this model reveals that it can explain 93.5% of the variance in activity. The *Q*^2^_LOO_ value of 0.899 was much larger than 0.5, indicating that the developed model had very good stability and predictive ability. In addition, the value of *R*^2^_test_ for the external prediction was 0.902, showing the good prediction and generalization ability.

Finally, to confirm the robustness of the model, the Y-randomization test was performed in this study. The dependent variable vector is randomly shuffled and a new model is constructed. If the new model gives significantly lower values for both *R*^2^_train_ and *Q*^2^_LOO_ statistics compared to the original model, the original generated model is not considered as resulting from a chance correlation [18]. The results of ten Y-randomization tests are summarized in Table 5. As can be seen, all new *R*^2^_train_ and *Q*^2^_LOO_ values were much lower than those of the original model. Thereby, the good results for the MLR model were not due to a chance correlation or structural dependency of the training set.

#### 2.1.2. Model Applicability Domain Analysis for the MLR Model

Finally, to evaluate the generalization degree of the generated model, the applicability domain (AD) was defined by a Williams plot. In the Williams plot, leverage values *versus* standardized residuals were plotted to detect both the structurally influential chemicals (*X* outliers) and the response outliers (*Y* outliers) [19]. The leverage value h is defined as:

(1) $$h_{i}=x_{i}^{T}{\left(X^{T}X\right)}^{-1}x_{i}\;\left(i=1,\;2,\;\ldots ,\;n\right)$$

where $x_{i}$ is the descriptor row vector of compound, *X* is the matrix of the descriptor values of the training set and n is the number of training sets. The superscript “*T*” refers to the transposed value of the matrix/vector [19,20]. When a leverage value *h* is higher than the threshold value h* (calculated as 3(*m* + 1)/*n*, where m is the number of model parameters and n is the number of the training set), it is considered as *X* outliers. In addition, a value of ±3.0 standard deviation units is widely used as a cut off value for defining *Y* outliers.

**Figure 2:**
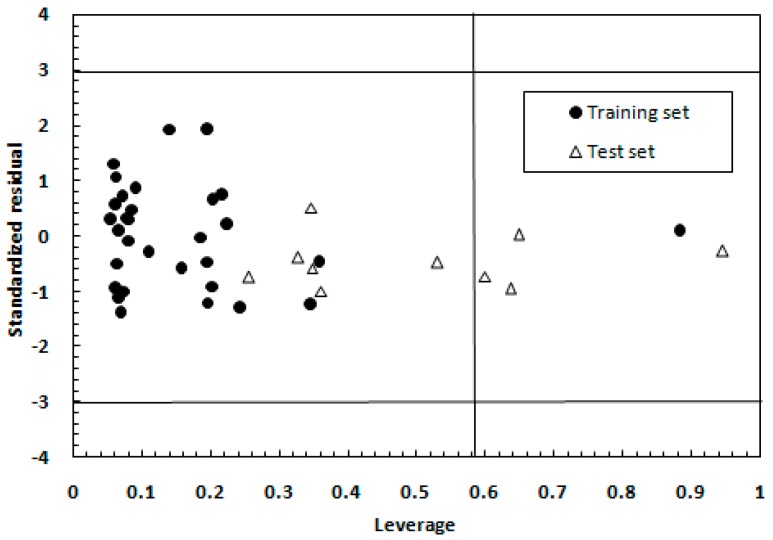
The Williams plot for the MLR model.

In this study, the Williams plot for the MLR model is shown in Figure 2. From this Figure 2, it can be found that no *Y* outliers existed in the investigated data set. Nevertheless, there were five molecules (compound 18, 20, 32, 37 and 26) with a leverage value higher than the warning leverage limit (0.581), but their predicted values were very satisfactory, with standard residuals lower than ±1.0 standard deviation units. Hence, these molecules were not influential in the fitting performance of the model. Conversely, it further showed the reliability of the predictions of the generated model as well as confirmed its good generalization ability [19]. Therefore, compounds with high value of leverage and good fitting in the developed model can stabilize the model, and not be considered as *X* outliers.

As can be seen from the above results, the MLR model was significantly highly predictive, reliable and robust. It can be used to predict the FXR agonist activity of new AAD.

#### 2.1.3. Interpretation of the Descriptors

The MLR model encompassed five descriptors: b_rotN, RPC^−^, opr_leadlike, SlogP_VSA2 and ASA, indicating some vital physicochemical features of AAD to govern the FXR agonist activity. The relative importance of the descriptors in the model was varied in view of their standardized regression coefficients shown in Table 1 [19]. Therefore, the relative importance order is ASA > SlogP_VSA2 > b_rotN > RPC^−^ > opr_leadlike. ASA is the water accessible surface area calculated using a radius of 1.4 A for the water molecule. This showed that the water accessible surface area of FXR agonists might influence their agonist activity. Its positive coefficient value indicated that high polar groups tend to increase the agonist activity. For instance, it can be observed from the agonist activity of compounds 9 (having 3-cyanophenyl with pEC_50_ = 6.638) and 10 (having 3-methoxyphenyl with pEC_50_ = 6.420) or 1 (having 3-carboxyphenyl with pEC_50_ = 6.553) and 8 (having acetylphenyl with pEC_50_ = 6.319) in Table 3. Slogp_VSA2 is the subdivided surface area descriptor, which is based on the sum of the approximate accessible van der Waal’s surface area, calculated for each atom with contribution to the log of the partition coefficient (octanol/water) in the range of (−0.2,0). Its negative coefficient value indicated that high hydrophobicity tended to decrease the agonist activity. Obviously, the bioactivity of molecules with aliphatic chains at region A in Table 3, such as 34 (with pEC_50_ of 5.602) or 35, 36, 37 and 38 (with pEC_50_ of 5.066–5.357), was lower than those without aliphatic chains such as 1 (with pEC_50_ of 6.553) or 39 and 40 (with pEC_50_ of 5.824–6.000). B_rotN is the number of rotatable single bonds. Its positive coefficient illustrated that more b_rotN was favorable to the FXR agonist activity. For instance, the bioactivity of compounds 15 and 16 or 35, 36 and 38 are varied in order: 16 (having b_rotN of 10) > 15 (having b_rotN of 9) or 38 (having b_rotN of 11) > 36 (having b_rotN of 10) > 35 (having b_rotN of 9). RPC^−^ is a relative negative partial charge descriptor that depends on the partial charge of each atom of a chemical structure. The positive sign of this descriptor illustrated that the relative negative partial charge of the molecule was favorable for the agonist activity. It can be quickly understood by comparing molecules 13 (having –CONH_2_ headgroup with pEC_50_ = 7.131) and 1 (having –COOH headgroup with pEC_50_ = 6.553). Opr_leadlike belongs to atom count and bond count descriptors that refer to the number of violations of the Oprea’s lead-like test. The positive contribution of this descriptor indicated that the high value of opr_leadlike was beneficial to the bioactivity.

Therefore, high polar groups such as the acidic headgroup together with high values of RPC^−^ and b_rotN are favorable for FXR agonist activity. Further, the aliphatic chain has a negative effect on it.

### 2.2. 3D-QSAR Models

#### 2.2.1. CoMFA Analysis

To graphically visualize the key chemical structural features that attributed to enhance the FXR agonist activity, CoMFA models were derived. The results of the CoMFA studies are listed in Table 4. The optimum number of components and filtering value for the CoMFA models were four and six, which were calculated by selecting the highest *Q*^2^_LOO_ value. The generated CoMFA model illustrated a *Q*^2^_LOO_ value of 0.802 (>0.5) by four components (*RMSE_LOO_* = 0.383). The non-cross-validated PLS analysis with the four components resulted in *R*^2^_train_ of 0.944, F value of 109.711 and *RMSE*_train_ of 0.203 and *R*^2^_test_ of 0.892. The contributions of steric and electrostatic fields calculated by the CoMFA model were 49.7% and 50.3% of the variance, respectively. The obtained high *R*^2^_train_, *Q*^2^_train_ and *F* values along with the lower *RMSE*_train_ indicated the satisfactory predictive ability of the derived model (Table 4). The pEC_50_ values predicted by the CoMFA model are listed in Table 3. Figure 1B demonstrates the correlation between experimental and predicted pEC_50_ values by the CoMFA model.

#### 2.2.2. CoMFA Contour Maps

**Figure 3:**
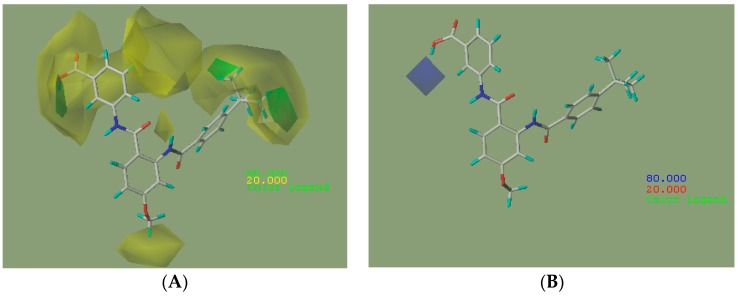
Contour maps of the CoMFA model: (**A**) steric field based on compound 30; (**B**) electrostatic field based on compound 30. Color values specify the CoMFA field levels that enclose volumes within which increase or decrease in bulk or positive charge favor higher dependent values.

**Figure 4:**
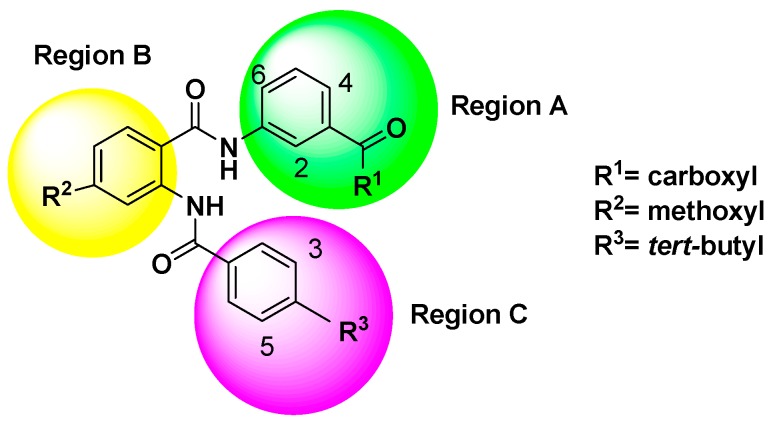
Structure of template compound (compound 30). The three regions A, B and C are depicted.

The steric and electrostatic contour maps derived by the CoMFA model based on the reference molecule (compound 30) are shown in Figure 3. The steric interactions are represented by green and yellow contours, while electrostatic interactions are represented by red and blue contours. In the green region of the steric contour plot, bulky substitutes enhance biological activity, while in the yellow regions, these are likely to decrease the activity. Blue contours represent regions where positive charge increases activity, whereas red-colored regions represent areas where negative charge enhances activity [17]. The three regions A, B, and C of compound 30 are depicted in Figure 4.

As shown in Figure 3A, there are two large yellow contours near regions A and C, one medium yellow contour near region B and one small yellow contour near region A, indicating that the bioactivity of molecules would be influenced by the introduction of bulky groups near these regions. According to Table 3, this can be explained by a comparison between molecules 21 (having –CH_3_ group at position 4 of region A with pEC_50_ = 7.347) and 32 (having –OCH_3_ at position 4 of region A with pEC_50_ = 7.328). The small yellow contour near position 6 of region A also suggested that the agonist activity would be decreased by the introduction of bulky groups here, such as compounds (18, 20, 19, 1) where the use of bulky groups (–OCH_3_ > –Cl > –F > –H) resulted in lower pEC_50_ values (5.328 < 5.959 < 6.319 < 6.553). A medium yellow contour near R^2^ at region B indicated that bulky groups here would cause lower activity. This can be observed by the comparison of molecules 27 (substituted by –CH_3_ with pEC_50_ value of 7.367) and 32 (substituted by –OCH_3_ with pEC_50_ value of 7.060), where the volume of –CH_3_ was smaller than –OCH_3_. This can also be observed by a comparison of compounds 25 (substituted by –Cl with pEC_50_ value of 7.328) and 29 (substituted by –Br with pEC_50_ value of 7.319). Another large yellow contour near region C showed that bulky groups at positions 3 and 5 of region C would lead to lower activity. For instance, the agonist activity of compounds 35 or 40 (substituted by naphthalen-2-yl) and 34 or 1 (substituted by 4-*tert*-butylphenyl) was varied in the order: 35 < 34 or 40 < 1. One small green contour near positions 2 and 3 of region A indicated that FXR agonist activity would be enhanced by introduction of bulky groups here. This can be observed by comparing molecules 17 (having –CH_3_ group at position 2 of region A) and 1 (having –H group at position 2 of region A), where using a bulky group influenced the outcome of pEC_50_ values (7.377 > 6.553). This can also be observed by comparing the bioactivity of molecules 16 and 15, where using bulky groups (–CH_2_CH_2_COOH > –CH_2_COOH) at position 3 of region A lead to higher pEC_50_ values (7.194 > 6.377). Another two small green contours near R^3^ substituent groups at region C showed the favorable effect of bulky groups here in increasing the biological activity of compounds. For instance, this can be explained by comparing the activity of compounds 1 (substituted by –C(CH_3_)_3_ with pEC_50_ value of 6.553) and 2 (substituted by –CF_3_ with pEC_50_ value of 5.161) or compounds 34 (substituted by –C(CH_3_)_3_ with pEC_50_ value of 5.602) and 33 (substituted by –CH_2_CH_3_ group with pEC_50_ value of 5.237).

As can be seen from Figure 3B, there was one medium blue contour near positions 2 and R^1^ of region A, which showed the favorable effect of electro-donating groups in increasing the biological activity of compounds. For instance, it can be observed by molecules 17 (having –CH_3_ group at position 2 of region A with pEC_50_ value of 7.377) and 1 (having –H group at position 2 of region A with pEC_50_ value of 6.553). This also can be explained by comparing the activity of compounds 13, 1 and 8, where using electro-donating substituents at R^1^ (–NH_2_ > –OH > –CH_3_) would result in higher pEC_50_ values (7.131 > 6.553 > 6.319).

### 2.3. Design of New Partial FXR Agonists

#### 2.3.1. Chemical Structure Design

According to the information derived from the contour maps generated by the 3D-QSAR models, some important information about the chemical structures requirement was presented to investigate the effect of each kind of group as the substituent for regions A, B and C on FXR agonist activity. The bulky groups with lower electronegativity at positions 2 and R^1^ substituent of region A together with bulky groups at R^3^ of region C were considered to enhance the FXR agonist activity. However, the presence of bulky groups at positions 4 and 6 of region A, R^2^ of region B and 3 and 5 of region C would decrease the agonist activity. Therefore, some new compounds as potent FXR agonists were designed and are listed in Table 6. To investigate the results of each substituent on the activity results, CoMFA was the best modeling tool for use. The newly designed compounds showed that the bulky groups with lower electronegativity at R^1^ of region A had positive effects. This can be observed by comparing compound N3 (having –N(CH_3_)_2_ at R^1^ of region A with predicted pEC_50_ value of 8.322) with template compound T30 (having –OH at R^1^ of region A with predicted and actual pEC_50_ values of 8.175 and 8.097, respectively). The next attempt was to improve the effects of functional groups at R^2^ of region B where bulky effects were presented. It was observed that the addition of less bulky groups (such as –CH_3_) at R^2^ of region B (Table 6) can lead to better agonist activity (compound N2 with predicted pEC_50_ value of 8.323). Then, to investigate the bulky effects at R^3^ of region C, different bulky groups were tried. This can be observed by comparing compounds N1, N2, N4 (having –C(CF_3_)_3_, –C(CH_3_)_3_ and –CI_3_ at R^3^ of region C with predicted pEC_50_ values of 8.350, 8.323 and 8.304, respectively). Finally, to observe the effects of the addition of lower electronegativity groups at positions 2 of region A, the donor groups were investigated. It can be seen that using donor group substituent (–H < –CH_3_ < –OH) at R^2^ of region A would lead to an increase in the predicted pEC_50_ values in the compounds: N7 (having –CH_3_ with pEC_50_ = 8.374), N9 (having –OH with pEC_50_ = 8.388) and N1 (having –H with pEC_50_ = 8.350). Among the designed compounds, N9 presented the highest activity with a pEC_50_ value of 8.388. To understand the origin of this increase in activity, compounds T30 and N3 can be compared.

#### 2.3.2. Molecular Docking Study

These molecules were ideally best based on their chemical structures, physicochemical properties and biological activity. Hence, molecular docking embedded in Molecular Operating Environment (MOE2008.10, Chemical Computing Group, Montreal, QC, Canada) was applied to study the binding modes and important interactions.

Prior to the docking, the crystal structure of FXR complexed with benzimidazole-based partial agonistic ligand was first downloaded from a protein data bank (PDB: 3OLF). The protein was protonated using the AMBER99 force field. A set of possible conformations of nine newly designed molecules was prepared by the conformational generation function of MOE. Then, molecular docking was carried out by following parameters: the binding site was defined by the ligand atom mode; triangle matcher was used as a placement method; two rescoring methods were computed, rescoring 1 was selected as London dG; rescoring 2 was selected as affinity; force field was used as a refinement [21].

**Figure 5:**
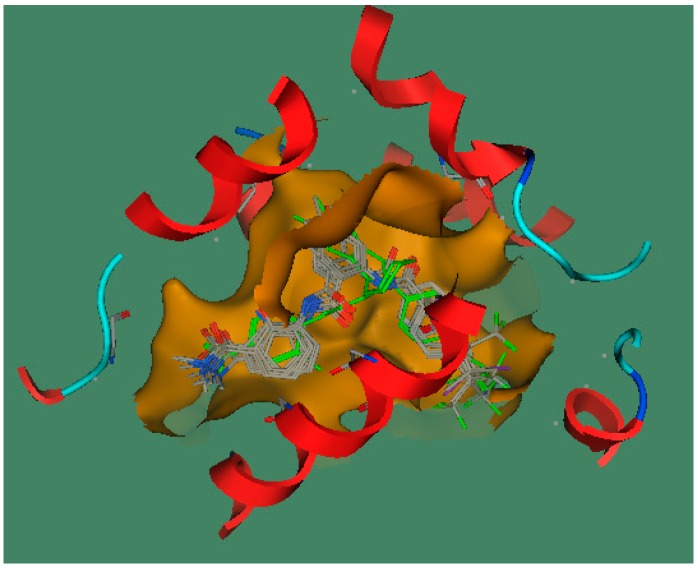
The best docked conformations and poses of newly designed compounds in the ligand binding domain of FXR.

**Figure 6:**
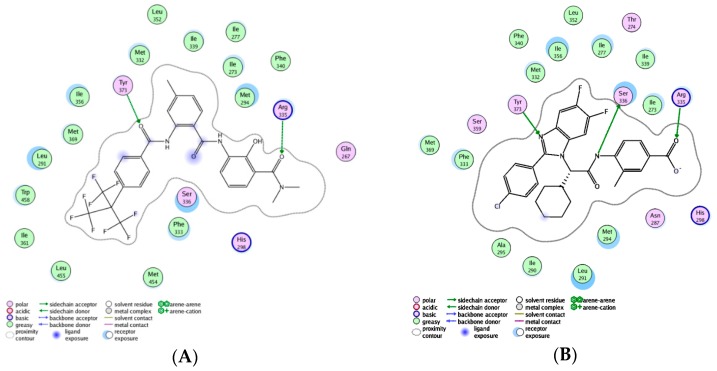
The 2D representation of docking of compounds N9 (**A**) and complexed ligand (**B**) into the FXR active site.

A critical factor that determines the effectiveness of a docking program is its ability to reproduce ligand poses in the receptor as close to those found in X-ray deduced structures as possible [22]. In this docking study, the root-mean-square distance (RMSD) parameter measured between the complexed ligand and the redocked ligand was 0.5749 Å, suggesting that the docking results were very suitable and reliable. Docking results are listed in Table 6. Obviously, these newly designed compounds had higher docking scores for FXR than the known template compound T30, which was in agreement with the 2D- and 3D-QSAR results. The best docked orientation of compounds is shown in Figure 5, showing that newly designed compounds can be well docked into the ligand binding site of FXR. The best docked conformation of the most active compound N9, as shown in Figure 6A, revealed that the presence of perfluoroalkyl chain substituted groups at region C allowed for potentiation of strong hydrophobic interactions with Met369, Leu291, Trp458, Met454, Ile361, Leu455 and Phe333 in the active site of FXR and formed two H-bonds with Arg335 and Tyr373. Comparative molecular docking between compound N9 and the complexed ligand, shown in Figure 6, indicated that the former had a better binding score than the latter, suggesting that hydrophobic interactions between groups at region C with these amino acids played a dominant role in the FXR agonist activity. Thereby, the hydrophobic interaction of groups at region C seems to stabilize the compound within the binding site, thus contributing greater activity.

## 3. Experimental Section

### 3.1. Data Set

The structures and biological activities of 41 AAD as FXR agonists used for the QSAR analyses were taken from Merk *et al.* [8,9] and are listed in Table 3. The agonist activity (EC_50_) value was converted to a logarithmic-scale pEC_50_ value, which was taken as the dependent parameter for the QSAR study. In order to establish a reliable model, the data set was randomly divided into two subsets, a training set of 31 compounds (approximately 75% of the data) that represented a wide range of varied structures and a test set of 10 compounds (approximately 25%) that followed the distribution of the activity values for the training set [14]. The training set is to build models, while the test set marked by the asterisk in Table 3 will be used to evaluate the prediction ability of the final training set model.

### 3.2. 2D-QSAR Studies

#### 3.2.1. Descriptors Calculation

All 2D structures of the molecules in Table 3 were sketched and their 3D structures were subjected to energy minimization using the molecular mechanics force field (MMFF) method with a convergence criterion of 0.01 kcal/mol and partial atomic charges. The final geometry optimization of each energy-minimized structure was carried out by stochastic conformational search. Then, only the lowest energy conformer of each structure was used to calculate 327 descriptors by employing the QuaSAR module of MOE [23]. These calculated descriptors include three classes: 2D descriptors, which use the atoms and connection information of the molecules, internal 3D (i3D), which uses 3D coordinate information about each molecule and external 3D (x3D), which uses 3D coordinate information with an absolute frame of reference. All the above processes were performed using MOE2008.10 package.

#### 3.2.2. Stepwise Multiple Linear Regression (SW-MLR)

In this study, a stepwise technology combined with MLR (SW-MLR) was employed to select a set of the most relevant descriptors for model constructions. The stepwise regression combines forward and backward selections. It selects statistically meaningful variables that can appreciably increase the residual sum of squares checked by the Fisher test [24]. Therefore, different MLR models will be derived in this procedure. The selection of a good MLR equation is made by statistical parameters such as the squared correlation coefficient (R^2^), root mean standard error (RMSE), and Fisher statistic [25]. The best MLR model should have high R^2^ and Fisher statistic, and low RMSE.

### 3.3. 3D-QSAR Studies

#### 3.3.1. Molecular Alignment

**Figure 7:**
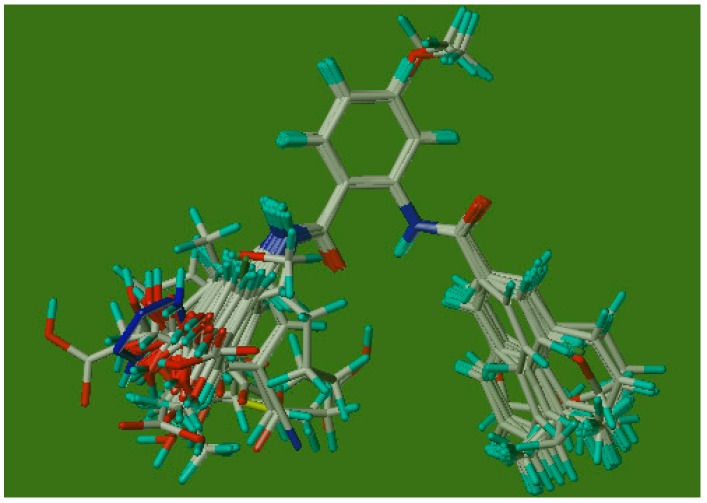
Alignment of training and test set compounds on compound 30. Baby blue, red, blue, green, gray and yellow signify hydrogen atom, oxygen atom, nitrogen atom, fluorine atom, carbon atom and sulfur atom, respectively.

The 3D-QSAR model was constructed by CoMFA embedded in SYBYL 6.9. Because the prediction accuracy of CoMFA is highly dependent on the structural alignment of the molecules with a reference compound, the selection of the template molecule plays an important role in performing CoMFA. Generally, the lowest energy conformer of the most active compound is selected as a template molecule for superimposition of all other compounds [26]. Therefore, compound 30 (as shown in Figure 4) was identified as a reference molecule in view of its highest activity, and all of the remaining compounds were aligned on it to derive CoMFA models. The structures of the aligned molecules are demonstrated in Figure 7.

#### 3.3.2. CoMFA Modeling

After aligning the molecules within the lattice that extended 4 Å units beyond the align molecules in all directions, an *sp*^3^ hybridized carbon was utilized as a probe atom to generate the steric and electrostatic fields with a charge of +1.0 and a van der Waals radius of 1.52 Å. The steric and electrostatic contributions were set as a default cut-off energy value of 30 kcal/mol. A partial least-squares (PLS) method, an extension of multiple regression analysis, was applied to calculate the minimal set of grid points and then linearly correlate the CoMFA fields to the pEC_50_ values in order to generate the CoMFA model [27].

### 3.4. Model Validation

The predictive ability and reliability of 2D- and 3D-QSAR models were evaluated by internal and external validations. The leave-one-out (LOO) cross-validation technology is often considered as the best way to internally validate the quality of derived models [28]. The LOO produces a number of models by deleting one object from the training set, which employs all the information available. Generally, when the value of LOO crossed validated correlation coefficient (*Q*^2^_LOO_) goes over a threshold value of 0.5, the model is acceptable [29]. In addition, external validation is also essential and significant to evaluate the generalization performance of the proposed model [25,30]. The statistical parameters, such as the root mean square errors (*RMSE_tes_*_t_) and the squared correlation coefficient (*R*^2^_test_) of the external test set were calculated to assess the performance of the model [31].

All algorithms were written in MATLAB 8.0 and run on a computer [Intel(R) Pentium(R), 2.00-GB RA].

## 4. Conclusions

In this paper, a three level *in silico* approach was applied to investigate some important structural and physicochemical aspects of highly potent partial FXR agonists. Initially, 2D-QSAR using methods of both SW-MLR and 3D-QSAR CoMFA studies was performed based on forty-one AAD. Satisfactory results were obtained with the proposed methods. The best derived 2D-QSAR model by SW-MLR can explain 93.5% of the variance in activity with a low RMSE of 0.219. In addition, the 2D-QSAR study demonstrated that b_rotN, RPC^−^, opr_leadlike, SlogP_VSA2, ASA of molecules had high correlation with the FXR agonist activity. Meanwhile, the best 3D-QSAR model presented higher predictive ability (*R*^2^_train_ = 0.944, *RMSE*_train_ = 0.203, *Q*^2^_LOO_ = 0.802, *R*^2^_test_ = 0.892) compared with the 2D-QSAR models. The derived contour maps from the 3D-QSAR model suggested the significant structural features (steric and electronic effects) required for improving biological activity. Consequently, the bulky groups with lower electronegativity at positions 2 and R^1^ substituent of region A together with bulky groups at R^3^ of region C were considered to enhance the FXR agonist activity. However, the presence of bulky groups at positions 4 and 6 of region A, R^2^ of region B and 3 and 5 of region C would decrease the agonist activity. Therefore, the obtained 2D- and 3D-QSAR models could provide valuable guidance for future design of new potent partial FXR agonists with an anthranilic acid skeleton in the drug discovery process. Finally, nine newly designed AAD with predicted pEC_50_ values higher than the known template compound were docked to the ligand binding domain of FXR. The molecular binding patterns and docking scores of these nine molecules also suggested that they can be robust and potent partial FXR agonists in agreement with the QSAR results. This also revealed that the hydrophobic interaction of groups at region C with Met369, Leu291, Trp458, Met454, Ile361, Leu455 and Phe333 seemed to stabilize the compound within the binding site, thus contributing greater activity. To the best of our knowledge, this work constituted the first *in silico* study for AAD as partial FXR agonists.

## Figures and Tables

**Table 1:** Selected descriptors of multiple linear regression.

Descriptor	Chemical Meaning	Coefficient	VIF	Stand Coefficient
b_rotN	Number of rotatable single bonds	0.362	2.888	0.408
RPC^−^	Relative negative partial charges	14.001	1.171	0.393
opr_leadlike	One if and only if the number of violations of Oprea‘s lead-like test <2 otherwise zero	0.318	1.271	0.136
SlogP_VSA2	The subdivided surface area descriptor, which is based on sum of the approximate accessible van der Waal’s surface area	−0.049	3.728	−0.567
ASA	Water accessible surface area calculated using a radius of 1.4 A for the water molecule	0.016	1.539	0.848
Constant	-	−9.717	–	–

**Table 2:** The correlation matrix of descriptors.

Descriptor	b_rotN	RPC^−^	opr_leadlike	SlogP_VSA2	ASA
b_rotN	1.000	0.359	0.137	0.715	−0.032
RPC^−^	0.359	1.000	−0.010	0.215	−0.289
opr_leadlike	0.137	−0.010	1.000	0.298	−0.461
SlogP_VSA2	0.715	0.215	0.298	1.000	−0.366
ASA	−0.032	−0.289	−0.461	−0.366	1.000

**Table 3:** Molecular structures and corresponding experimental and predicted pEC_50_ values of the AAD as partial FXR agonists.

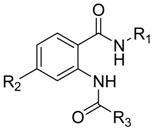							
NO.	R_1_	R_2_	R_3_	EC_50_ (µM)	pEC_50_	2D-Pred	3D-Pred
						SW-MLR	CoMFA
1	3-carboxyphenyl	H	4-*tert*-butylphenyl	0.28	6.553	6.784	6.704
2 *	3-carboxyphenyl	H	4-(trifluoromethyl)phenyl	6.9	5.161	5.653	5.171
3	3-carboxyphenyl	H	4-bromophenyl	3.7	5.432	5.451	5.485
4	3-carboxyphenyl	H	benzo[*d*][1,3]dioxol-5-yl	10	5.000	5.336	5.133
5 *	3-carboxyphenyl	H	2,3-dihydrobenzo[*b*][1,4]dioxin-6-yl	4.9	5.310	5.714	5.133
6	3-carboxyphenyl	H	3-fluoro-4-(trifluoromethyl)phenyl	5	5.301	5.232	5.358
7 *	3-carboxyphenyl	H	styryl	5.2	5.284	4.850	5.639
8	3-acetylphenyl	H	4-*tert*-butylphenyl	0.48	6.319	6.592	6.775
9	3-cyanophenyl	H	4-*tert*-butylphenyl	0.23	6.638	6.619	6.506
10	3-methoxyphenyl	H	4-*tert*-butylphenyl	0.38	6.420	6.661	6.327
11	3-(methylthio)phenyl	H	4-*tert*-butylphenyl	0.2	6.699	6.616	6.655
12	3-(1*H*-tetrazol-5-yl)phenyl	H	4-*tert*-butylphenyl	2.9	5.538	5.854	5.695
13	3-carbamoylphenyl	H	4-*tert*-butylphenyl	0.074	7.131	7.203	7.099
14 *	3,4-bimethoxyphenyl	H	4-*tert*-butylphenyl	0.071	7.149	7.771	6.606
15	3-(carboxymethyl)phenyl	H	4-*tert*-butylphenyl	0.42	6.377	6.493	6.414
16	3-(2-carboxyethyl)phenyl	H	4-*tert*-butylphenyl	0.064	7.194	7.143	6.999
17	2-methyl-3-carboxylphenyl	H	4-*tert*-butylphenyl	0.042	7.377	7.115	7.266
18	2-methoxy-5-carboxyphenyl	H	4-*tert*-butylphenyl	4.7	5.328	5.307	5.444
19	2-fluoro-5-carboxyphenyl	H	4-*tert*-butylphenyl	0.48	6.319	6.148	6.361
20*	2-chloro-5-carboxyphenyl	H	4-*tert*-butylphenyl	1.1	5.959	6.569	5.759
21	3-carboxy-4-methylphenyl	H	4-*tert*-butylphenyl	0.045	7.347	7.209	6.934
22 *	3-carboxy-4-methoxylphenyl	H	4-*tert*-butylphenyl	0.047	7.328	7.650	7.563
23	3-carboxy-4-chlorophenyl	H	4-*tert*-butylphenyl	0.28	6.553	6.845	6.931
24	3-carboxy-4-bromophenyl	H	4-*tert*-butylphenyl	0.15	6.824	6.937	6.889
25	3-carboxyphenyl	chloro	4-*tert*-butylphenyl	0.047	7.328	6.862	6.868
26 *	4-carboxymethylphenyl	H	naphthalen-2-yl	3.1	5.509	5.492	5.082
27	3-carboxy-4-methylphenyl	methyl	4-*tert*-butylphenyl	0.043	7.367	7.048	7.510
28	3-carboxyphenyl	methyl	4-*tert*-butylphenyl	0.061	7.215	7.334	6.942
29	3-carboxyphenyl	bromo	4-*tert*-butylphenyl	0.048	7.319	7.160	7.241
30	3-carboxyphenyl	methoxy	4-*tert*-butylphenyl	0.008	8.097	7.888	8.175
31	3-carboxy-4-methylphenyl	chloro	4-*tert*-butylphenyl	0.11	6.959	7.178	7.027
32 *	3-carboxy-4-methylphenyl	methoxy	4-*tert*-butylphenyl	0.087	7.060	7.861	6.858
33	3-carboxypropyl	H	4-ehylphenyl	5.8	5.237	5.058	5.253
34	3-carboxypropyl	H	4-*tert*-butylphenyl	2.5	5.602	5.748	5.885
35	3-carboxypropyl	H	naphthalen-2-yl	8.6	5.066	4.598	4.817
36	4-carboxybutyl	H	naphthalen-2-yl	8.3	5.081	5.086	4.795
37 *	3-methoxy-3-oxopropyl	H	naphthalen-2-yl	7.1	5.149	5.377	4.857
38	5-carboxypentyl	H	naphthalen-2-yl	4.4	5.357	5.657	5.416
39	4-carboxyphenyl	H	naphthalen-2-yl	1.0	6.000	5.882	5.902
40	3-carboxyphenyl	H	naphthalen-2-yl	1.5	5.824	5.750	5.990
41 *	4-carboxybenzyl	H	naphthalen-2-yl	1.3	5.886	6.722	5.405

* denotes the test set compounds.

**Table 4:** Statistical parameters obtained using the MLR and CoMFA models.

Model	Training Set					Test Set	
	*R*^2^_train_	*RMSE*_train_	*F*	*Q*^2^_LOO_	*RMSE*_LOO_	*R*^2^_test_	*RMSE*_test_
MLR	0.935	0.219	72.353	0.899	0.299	0.902	0.534
CoMFA-1	0.944	0.203	109.711	0.802	0.383	0.892	0.330

**Table 5:** *R*^2^_train_ and *Q*^2^_LOO_ values after Several Y-randomization tests.

No.	1	2	3	4	5	6	7	8	9	10
***R*^2^_train_**	0.150	0.145	0.188	0.08	0.123	0.179	0.144	0.186	0.141	0.131
***Q*^2^_LOO_**	0.013	0.058	0.016	0.039	0.014	0.012	0.105	0.000	0.005	0.014

**Table 6:** Chemical Structures of Newly Designed partial FXR agonists based on 3D-QSAR Models.

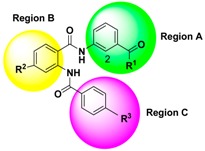							
Name	R^1^	Substituents at Position 2	R^2^	R^3^	Predicted pEC_50_ Values		Docking Scores
					SW-MLR	CoMFA	
T30	OH	H	OCH_3_	C(CH_3_)_3_	7.888	8.175	−10.176
N1	N(CH_3_)_2_	H	CH_3_	C(CF_3_)_3_	9.032	8.350	−14.053
N2	N(CH_3_)_2_	H	CH_3_	C(CH_3_)_3_	8.274	8.323	−11.081
N3	N(CH_3_)_2_	H	OCH_3_	C(CH_3_)_3_	8.626	8.322	−10.716
N4	N(CH_3_)_2_	H	CH_3_	CI_3_	8.744	8.304	−12.038
N5	N(CH_3_)_2_	OH	CH_3_	C(CH_3_)_3_	8.260	8.360	−11.602
N6	N(CH_3_)_2_	OH	CH_3_	CI_3_	8.828	8.357	−12.073
N7	N(CH_3_)_2_	CH_3_	CH_3_	C(CF_3_)_3_	9.123	8.374	−14.193
N8	N(CH_3_)_2_	NH_2_	CH_3_	C(CF_3_)_3_	8.816	8.378	−14.335
N9	N(CH_3_)_2_	OH	CH_3_	C(CF_3_)_3_	9.024	8.388	−14.347

R^1^_,_ R^2^, R^3^ represent substituent groups, respectively.
